# A Gum-Chewing Variety

**Published:** 1885-07

**Authors:** C. M. Wright

**Affiliations:** Cincinnati, O.


### A GUM-CHEWING VARIETY.

BY C. M. WRIGHT, CINCINNATI, O.

In a three-minute paper read before the Mississippi Valley Dental Society, March 5th, 1885, I suggested that if the "Development Hypothesis" of scientists be true, then we might, perhaps, by taking notice of it, be able to obtain a broader view of some of the laws influencing the destruction of the human teeth. The paper was effectually "sat upon" by prominent gentlemen, who slightingly referred to it in the discussion which followed; yet from all parts of the room, and during the entire discussion, vague hints on the value of Chewing-gum as a means of improving the organs of mastication, were constantly flying about. Now upon what other ground than that of the Development Hypothesis that I referred to can the members of the Mississippi Valley Dental Society base a theory of the value of Chewing-gum? Although the profession at large does not care much for philosophy, it accepts, of course, the influence of the laws of inheritance, selection, development, etc., as applying with wonderful significance to the morphological peculiarities of the animal world in general. And while a few seem to resent the attempted application of these laws to man and to the teeth of man (so high an authority as Dr. J. Taft, at this same meeting above referred to, positively denied that these laws applied to man), still, when we talk about Chewing-gum in open and serious debate, we tacitly admit that a changed condition of the human jaws, viz.: from a partially passive condition caused by soft food and the lack of necessity for rigorous chewing or employment, to high activity caused by the use of chewing-gum and the fascinating necessity of chewing it like mad, will cause the organs to *immediately begin to undergo a*

*change in structure*, and this is the basis of the hypothesis we have referred to. But, as was stated at the meeting, we want to talk about *practical* things and practical methods. We prefer, too, to build and accept theories that have a practical, every-day sort of plausibility about them. We do not care particularly about the *facts*, if the *theory* is plausible. We do not want to worry our brains about principles or laws, if we can get ready-made theories that will explain, in a popular and agreeable way, notions which we think we can safely teach our patients. We have had a long run of oatmeal porridge, cracked wheat, and unbolted flour. The public has taken our doses of this unpalatable but healthy food *ad nauseam*. The joke about Scotch men and English horses is classic. We have put phosphate of lime, too, bodily into teeth, by assimilation and by peripheral absorption, although we have neglected scientific analysis of the teeth in the laboratory. We have not asked chemistry to take the tooth before and after taking phosphates, and to give us the pictures. We have not questioned the capacity of observation which has seen fire fly from a tooth when struck by an excavator, after only a few months' use of oatmeal porridge by the patient—a tooth which, before, was soft as butter. Theory has said, "the tooth contains now more phosphate of lime," and we have said it, morning, noon and night, ever since, without once reflecting upon the *facts*. The medical profession has shifted the phosphates, in rather a promiscuous way too, in the bones of her patients. Old men's bones break easier than children's bones, because old men's bones have more phosphates. Surgery says: "Young people's bones unite more readily after fracture than old people's bones, because there is less phosphate in young bones." "Pregnant woman's bones contain less phosphate of lime, because the phosphates are taken out of her bones to enter into and build up the bones of the fœtus."

These are our facts (?), which have become like axioms, notwithstanding the experiments of Chossat and others.*

*This observer determined by numerous nutritive experiments on birds, that by withholding the lime salts from their food, not only the mineral but the *animal* constituents of bone were uniformly diminished. Reference might also be made to the observations of Dr. Stark, who after numerous experiments concludes "that the amount of earthy matter in healthy bones is nearly uniform over the whole animal kingdom." Von Bibra also found that the proportions of animal and earthy matter are very nearly the same in a fœtal and adult bone.

But we, as a profession, and the people, too, are getting tired of these statements. We want something fresh, something startling, something popular, and plausible, and new; and what could be newer or more popular and plausible than the theory about chewing-gum? Throw away your oatmeal, and chew gum. If we can only prevent cranky individuals from raising the hue and cry, and pointing out some blood-poisoning or cancer-inviting qualities in Spruce or Tolu, we may safely and unthinkingly go down to the next generation—on chewing-gum. Happy thought! And here's your theory to back you. Constant exercise of teeth and jaws excites extra local blood supply; extra blood supply means extra nourishment; extra nourishment tends to increased vigor and development; increased vigor and development means better health and less disease, better resistance to acids and the entire discomfiture of the bacteria. But to get the full benefit of this we must encourage the development for some generations, and to encourage this we must study the laws of Inheritance and resort to artificial sexual selection. Our children must be encouraged to chew gum. Intelligence will direct us in this. Chewing tournaments, or matches, or rinks, can be established. Supervision can be exercised, and the best boy chewers and the best girl chewers can be gradually withdrawn from the feebler chewers, and made to associate together. The gum-chewing young man and the gum-chewing young woman will mate in time. During courtship they would chew gum. After marriage they would chew gum, especially during times of emotional excitement, and a proportion of their children would, we have every reason to believe, inherit a capacity and a tendency toward gum. Here, again, our supervising influence should come in. The most vigorous gum-chewers, those whose jaws showed the distinctive evidence of structural development, should be selected for association with other children of other gum-chewing parents, with like structural development, and the feeble chewers should be prevented, as far as moral and strategic influences go, from matrimony. I need not go on. Any one can imagine what a few generations would produce. We should undoubtedly have a variety or species of the human race with fine organs of mastication. Isn't this *practical* enough for the Mississippi Valley Dental Society?

But a word of warning. Let us be sure that this is the habit that will produce the variety before we risk the existence of our specialty. Therefore I call upon the scientific readers of the INDEPENDENT PRACTITIONER to busy and interest themselves in gathering the data of gum-chewing. Let us have evidence that gum-chewing will save teeth before we start our patients on the fascinating habit and begin the annihilation of ourselves as dentists.

*References.*—Herbert Spencer, Charles Darwin, Ernst Haeckel, Oscar Peschel, a late paper by Alex. Graham Bell upon "The Formation of a Deaf Mute Variety of the Human Race," Coleman's Ko-Ko Tolu, et al.

